# Relationships among patient characteristics, irradiation treatment planning parameters, and treatment toxicity of acute radiation dermatitis after breast hybrid intensity modulation radiation therapy

**DOI:** 10.1371/journal.pone.0200192

**Published:** 2018-07-16

**Authors:** Tsair-Fwu Lee, Kuo-Chiang Sung, Pei-Ju Chao, Yu-Jie Huang, Jen-Hong Lan, Horng-Yuan Wu, Liyun Chang, Hui-Min Ting

**Affiliations:** 1 Medical Physics and Informatics Laboratory of Electronics Engineering, National Kaohsiung University of Science and Technology, Kaohsiung, Taiwan, ROC; 2 Graduate Institute of Clinical Medicine, Kaohsiung Medical University, Kaohsiung, Taiwan, ROC; 3 Department of Radiation Oncology, Kaohsiung Chang Gung Memorial Hospital and Chang Gung University College of Medicine, Kaohsiung, Taiwan, ROC; 4 Department of Electrical Engineering, National Kaohsiung University of Science and Technology, Kaohsiung, Taiwan, ROC; 5 Department of Medical Imaging and Radiological Sciences, I-Shou University, Kaohsiung, Taiwan, ROC; North Shore Long Island Jewish Health System, UNITED STATES

## Abstract

To evaluate the relationships among patient characteristics, irradiation treatment planning parameters, and treatment toxicity of acute radiation dermatitis (RD) after breast hybrid intensity modulation radiation therapy (IMRT). The study cohort consisted of 95 breast cancer patients treated with hybrid IMRT. RD grade ≥2 (2^+^) toxicity was defined as clinically significant. Patient characteristics and the irradiation treatment planning parameters were used as the initial candidate factors. Prognostic factors were identified using the least absolute shrinkage and selection operator (LASSO)-based normal tissue complication probability (NTCP) model. A univariate cut-off dose NTCP model was developed to find the dose-volume limitation. Fifty-two (54.7%) of ninety-five patients experienced acute RD grade 2^+^ toxicity. The volume of skin receiving a dose >35 Gy (V_35_) was the most significant dosimetric predictor associated with RD grade 2^+^ toxicity. The NTCP model parameters for V_35Gy_ were TV_50_ = 85.7 mL and γ_50_ = 0.77, where TV_50_ was defined as the volume corresponding to a 50% incidence of complications, and γ_50_ was the normalized slope of the volume-response curve. Additional potential predictive patient characteristics were energy and surgery, but the results were not statistically significant.

To ensure a better quality of life and compliance for breast hybrid IMRT patients, the skin volume receiving a dose >35 Gy should be limited to <85.7 mL to keep the incidence of RD grade 2^+^ toxicities below 50%. To avoid RD toxicity, the volume of skin receiving a dose >35 Gy should follow sparing tolerance and the inherent patient characteristics should be considered.

## Introduction

Hybrid intensity modulation radiation therapy (hybrid IMRT) or IMRT after mastectomy or breast-conserving surgery is one of the major treatment options for breast cancer patients [1, 2]. The potential complications associated with these treatments can be reduced using modern treatment techniques and fractionation schemes. However, the skin is close to the target volume and naturally receives a high radiation dose [3]. The skin is relatively radiosensitive and may exhibit different degrees of damage after certain doses of radiation therapy (RT) [4]. Radiation dermatitis (RD) toxicities, such as skin erythema, breast edema, and breast fibrosis, are the most common complications after RT [5]. Therefore, physicians can expect RD to occur after RT. The degree of RD is related to the total radiation dose, the volume of the tissue irradiated, the proportion of the body irradiated, and the duration of the radiation dose received [6, 7]. The skin irradiated dose can be reflected on the isodose curve distribution during RT treatment planning.

The univariate normal tissue complication probability (NTCP) model can be used to describe the correlation between dosimetric parameters and the probability of RD. Skin dose-sparing parameters can be used by physicians to avoid RD damage and improve patient quality of life [8]. Previous studies showed that the quantitative analysis of normal tissue effects in the clinic could statistically estimate the dose tolerance of critical structures [9]. The grade of RD severity varies with the radiation dose. RD develops in a dose-dependent manner but the exact dose associated with different grades of RD is disputed within the literature. During standard radiation treatment, the first change to the skin is in the form of erythema. Ryan et al. (2012) showed that definite erythema occurs in the second or third week with an irradiation dose of 10~12 Gy [10], while Halperin et al. (2008) noted the occurrence of erythema with a dose of 20~40 Gy [11]. Dry desquamation appeared with an irradiation dose of 20~25 Gy in Ryan’s study, but not until a dose of 40~45 Gy was administered in the studies by Halperin and Washington [11, 12]. Moist desquamation sometimes occurred with an irradiation dose of 30~40 Gy in Ryan’s study, but was only seen with a dose above 45 Gy in Halperin’s study, and above 50 Gy in Washington’s work. These data show that the reported irradiation dose at which RD occurs differs among studies. Furthermore, erythema reportedly can occur at an irradiation dose of 6~40 Gy, dry desquamation at 20~45 Gy, and moist desquamation at 30~50 Gy. Accordingly, it is difficult to ascertain the safe irradiation dose limit. We are currently attempting to devise a convenient method to describe the relationship between dosimetric parameters and the risk of clinical RD. At present, we follow evidence-based guidelines for treating the local ethnic population where possible.

Skin complications may be affected by factors including the fractionation dose, the irradiated volume, the point dose, and beam energy. Furthermore, the treatment technique and patient-related factors such as age, height, body weight, tumor size, menopausal status, tumor markers, lymph node status, surgery, concurrent chemotherapy, and selected modalities can influence the severity of RD [3, 4, 13]. Multivariate NTCP modeling seeks to obtain maximal information regarding the correlation between inhomogeneous dose distributions and clinical patient parameters with corresponding RD outcome data in parametric models. Therefore, the clinical characteristics, risk factors, and dose-volume tolerances for RD after RT need to be considered to avoid RD toxicity after RT.

Considering the importance of these issues, the current study investigated the extent of RD and the incidence of predictive factors in breast hybrid IMRT patients, and also identified the NTCP dosimetric parameters for skin dose-sparing. Achieving a better understanding of the risk factors will help to improve treatment quality.

## Methods and materials

### Patient characteristics

A total of 95 breast cancer patients who were referred to our department for adjuvant irradiation between May 2010 and October 2013 were enrolled. All patients were treated with hybrid IMRT after breast conserving surgery (BCS) or modified radical mastectomy (MRM). The patients’ intrinsic characteristics and radiation therapy treatment planning parameters were analyzed. The patient characteristics are presented in Tables 1 and 2. The study was approved by the Chang Gung Memorial Hospital Institutional Review Board (103-1340B), and all experiments were performed in accordance with relevant international and national guidelines and regulations.

**Table 1:** Patients characteristics.

	Group 0 (n = 43) Value-x (%)	Group 1 (n = 52) Value-x (%)	*p* value
Age (y)			0.64
** **Mean	54.70	53.81	
** **Range	34.00–68.00	36.00–76.00	
** **<51	15 (16)	15 (16)	
** **51–60	15 (16)	25 (26)	
** **61–70	13 (13)	10 (11)	
** **>70	0 (0)	2 (2)	
BMI			0.65
** **Mean	24.51	24.95	
** **Range	17.60–40.35	17.35–41.10	
** **<21	10 (11)	9 (9)	
** **21–26	18 (19)	27 (28)	
** **>26	15 (16)	16 (17)	
Surgery			0.28
** **BCS	20 (21)	30 (32)	
** **MRM	23 (24)	22 (23)	
Chemotherapy			0.79
** **NO	16 (17)	18 (19)	
** **YES	27 (28)	34 (36)	
Tumor site			0.71
** **Left	19 (20)	25 (27)	
** **Right	24 (25)	27 (28)	
SCF			0.34
** **NO	32 (33)	34 (36)	
** **YES	11 (12)	18 (19)	
IMN			0.50
** **NO	37 (39)	42 (44)	
** **YES	6 (6)	10 (11)	
Smoking			0.68
** **NO	42 (44)	50 (53)	
** **YES	1 (1)	2 (2)	
AJCC Stage			
** **2	21 (22)	31 (33)	0.58
** **3	20 (21)	19 (20)	0.30
** **4	2 (2)	2 (2)	0.71
Photon Energy (MV)			
** **6	2 (2)	3 (3)	0.30
** **10	8 (8)	4 (4)	0.32
** **6&10	33 (35)	45 (48)	0.92
Radiation dermatitis (RD)			
** **Grade 1	43 (45)	0	
** **Grade 2	0	45 (48)	
** **Grade 3	0	7 (7)	

Abbreviations: Group 0: patients with dermatitis grade ≤1; Group 1: patients with dermatitis grade ≥2; SCF: Supraclavicular fossa; IMN: Internal mammary lymph nodes; AJCC: American Joint of Cancer Committee; Surgery: BCS: patients with breast conserving surgery; MRM: patients with modified radical mastectomy; Statistical significance was assumed at p < 0.05.

**Table 2:** Analyses of dosimetric parameters for patients without and with grade 2+ radiation dermatitis.

	Group 0 (n = 43)		Group 1 (n = 52)		*p* value
	Mean	Range	Mean	Range	
PTV (ml)	632.33	248.00–1499.00	739.26	217.00–1508.00	0.07
PTV-V_95%_ (%)	94.97	88.60–99.25	95.54	88.31–99.66	0.24
PTV-V_100%_ (%)	84.26	71.42–94.89	86.52	73.08–97.43	0.04
PTV-V_105%_ (%)	20.91	4.72–73.51	25.04	6.14–76.44	0.22
PTV-V_107%_ (%)	5.85	0.01–38.26	6.15	0.00–34.04	0.87
TV-V_107%_ (ml)	89.81	4.20–549.00	107.83	15.02–823.92	0.46
TV-V_110%_ (ml)	11.19	0.00–98.97	14.02	0.00–376.19	0.75
HI	1.12	1.05–1.23	1.11	1.01–1.18	0.34
CI	2.25	1.39–3.81	2.06	1.36–3.55	0.08
V_5 Gy_ (ml)	236.06	123.66–359.39	273.73	132.69–529.39	0.01
V_10 Gy_ (ml)	198.47	118.56–302.62	227.82	131.60–429.33	0.01
V_15 Gy_ (ml)	178.61	116.28–262.70	202.84	129.13–380.59	0.01
V_20 Gy_ (ml)	161.64	111.28–230.45	182.97	124.57–341.03	0.01
V_25 Gy_ (ml)	142.76	102.48–200.14	161.76	113.58–301.92	0.01
V_30 Gy_ (ml)	117.00	87.00–155.70	134.32	97.23–259.21	0.01
V_35 Gy_ (ml)	85.00	60.89–120.02	99.07	71.61–200.93	<0.01
V_40 Gy_ (ml)	47.32	27.69–76.16	57.25	33.72–130.90	0.01
V_45 Gy_ (ml)	15.26	5.71–33.47	20.63	5.72–63.72	0.03
V_50 Gy_ (ml)	1.30	0.02–6.52	3.66	0.03–34.94	0.13
V_52 Gy_ (ml)	0.27	0.00–2.59	1.61	0.00–21.09	0.21

Abbreviations: Group 0: patients with dermatitis grade ≤ 1; Group 1: patients with dermatitis grade ≥ 2; PTV: Planning Target Volume; PTV-Vx% = percent volume receiving X% of prescribed dose within PTV. Treated volume (TV) = the tissue volume which received the prescribed dose. TV-Vx% = percent volume receiving X% of prescribed dose within TV; HI: Homogeneity Index; CI: Conformity Index; Vx was defined as skin volume received X Gy, and X was 5, 10, 15, 20, 25, 30, 35, 40, 45, 50, and 52 Gy at the selected steps. Statistical significance was assumed at p < 0.05.

### Radiation treatment planning

A planning computed tomography (CT, Lightspeed RT16, GE Medical System, WI, USA) scan was obtained for each patient. The patients were immobilized using a thermoplastic cast and positioned on a breast board with both arms raised alongside their head. The treatment plans were created using the Philips Pinnacle^3^ treatment planning system (TPS) (version 9.2, Philips Medical Systems, Andover, MA, USA) that integrated an additional optimization engine (direct machine parameter optimization, DMPO) and a biological objective function based on generalized equivalent uniform dose (gEUD) processing. The plans were created using the 6/10-MV photon beams commissioned for an Elekta Precise™ Linac (Elekta, Crawley, UK) equipped with an 80-leaf 1-cm multi-leaf collimator. A collapsed cone convolution (CCC) algorithm was performed during convolution dose calculations to recover potential errors caused by the pencil beam convolution dose calculations used during optimization processing. Segment-weight optimization was also performed on the final segments [14, 15]. The plans were delivered in step-and-shoot mode.

The clinical target volume (CTV) was contoured on helical CT slices with a 3.75 mm slice thickness for each patient. The CTV was then expanded by 10 mm to create the planned target volume (PTV). According to the National Comprehensive Cancer Network Clinical Practice Guidelines (NCCN guidelines), the target volumes and organs at risk (OARs) (ipsilateral lung, contralateral lung, heart, and contralateral breast) were contoured at the time. If four or more axillary lymph nodes were positive, then the supraclavicular fossa (SCF) was irradiated; the internal mammary lymph nodes (IMN) were considered in patients with positive axillary nodes. A total dose of 5040 cGy in 28 fractions (180 cGy per day) was prescribed. The treatment was followed by a sequential electron boost to the tumor bed and scan from 14~20 Gy.

All patient treatments were planned with a four-field hybrid IMRT plan consisting of two open tangential fields and two IMRT fields using volume-based inverse planning. The tangential beams (a pair of IMRT tangents) were designed for use without wedges. The relative weights of the tangential beams were manually modified to achieve a dose coverage similar to that of the tangents plan [2, 16, 17]. The plans were optimized to cover the PTV and spare the surrounding OARs. The treatment was delivered using a single-energy 6 MV or 10 MV setting, and sometimes the combination of 6 MV and 10 MV. The energy selection was based on the patient chest wall separation.

### Chemotherapy

After surgical intervention for breast cancer, the decision regarding the need for adjuvant chemotherapy considered the risk of recurrence, toxicities, and comorbidities. For high-risk patients, cyclophosphamide methotrexate fluorouracil (CMF) or cyclophosphamide epirubicin 5-fluorouracil (CEF) were prescribed for a total of four to nine 4-week cycles. Four cycles of docetaxel and cyclophosphamide was an alternative regimen. In high-risk HER-2-positive disease, sequential chemotherapy with taxanes was administered concurrently with trastuzumab; trastuzumab was given for 1 year. Lower-risk, nodal-negative, HER-2-positive patients received paclitaxel and trastuzumab once a week for 12 cycles. The schedule and regimens were modified according to the patient’s clinical condition and the oncologist’s judgment as necessary.

### Endpoint evaluation

In this study, the skin volume was defined as the first 3 mm clipped from the skin surface around the treatment body. An auto-contouring tool was used to contour the entire skin volume. Although there is no gold standard for the measurement or management of RD toxicity, the same attending physician evaluated the severity of RD using Radiation Therapy Oncology Group (RTOG) Acute Radiation Morbidity Scoring Criteria in the last week of treatment. The grade of RD severity was defined as follows: 0, no change; 1, follicular, faint or dull erythema/epilation/dry desquamation; 2, tender or bright erythema, patchy moist desquamation; 3, confluent, moist desquamation other than skin folds; 4, ulceration. The endpoint RD was defined as patients with an RD grade toxicity ≥ 2 (2^+^) in this study.

### Patient characteristics and dosimetric parameters

The following candidate patient characteristic predictive factors were included in the variable selection procedure: age, body mass index (BMI), height, weight, surgery (BCS or MRM), T-boost (with/without tumor bed electron boost), chemotherapy, tumor site, SCF, IMN, smoking habits, TMN stage, and the delivery energy used.

To evaluate the dose effects on RD toxicity, several dosimetric parameters were analyzed as follows. V_X_ is defined as the skin volume that received X Gy, where X was 5, 10, 15, 20, 25, 30, 35, 40, 45, 50 and 52 at the selected steps.

The planning target volume (PTV-V), PTV-V_100%_, PTV-V_105%_, PTV-V_107%_ correspond to the percent volume receiving 100%, 105%, or 107% of the prescribed dose within the PTV. The treated volume (TV) is the tissue volume which received the prescribed dose. TV-X% describes the percent volume that received X% of the prescribed dose within the TV. The conformity index (CI) is the ratio of the PTV coverage to the prescription isodose volume in the treatment plans [18, 19], and was calculated as

$$CI=Vptv\times \frac{Vtv}{{\left(TVpv\right)}^{2}},$$

where V_TV_ is the treatment volume of the prescribed isodose, V_PTV_ is the volume of the PTV, and TV_PV_ is the volume of the V_PTV_ within the V_TV_. A CI value closer to one describes better conformal coverage. The homogeneity index (HI) evaluates the dose homogeneity in the PTV as follows[20]

$$HI=\frac{D5\%}{D95\%},$$

where *D*5% and *D*95% are the minimum doses delivered to 5% and 95% of the PTV. A higher HI indicates poorer homogeneity.

### Statistical analysis and NTCP modelling

A multivariate logistic dose-response NTCP model with LASSO was established to calculate the toxicity risk of RD; an explanatory-variable set was selected from 20 dosimetric and 10 clinical variables. Details regarding the multivariate logistic regression analysis have been described previously [21–23]. LASSO was performed with 10-fold cross validation as a regularization technique to select the optimal number of potential predictive factors for RD occurrence. The LASSO-based NTCP model used was reported previously [24–26]. After the predictive factors were selected, the system performance measures were verified using an area under the receiver operating characteristic curve (AUC), scaled Brier score, and Nagelkerke R^2^, Omnibus and Hosmer–Lemeshow tests.

The most significant dosimetric factor was used to develop a single mean-dose NTCP model. The parameters TV_50_ and γ_50_ used for the univariate NTCP regression model are shown for convenience in the curve fitting figure, where TV_50_ was defined as the volume corresponding to 50% incidence of complications, and γ_50_ was the normalized slope of the volume-response curve. Statistical analyses were performed using SPSS 19.0 (SPSS, Chicago, IL, USA).

## Results

**Fig 1:**
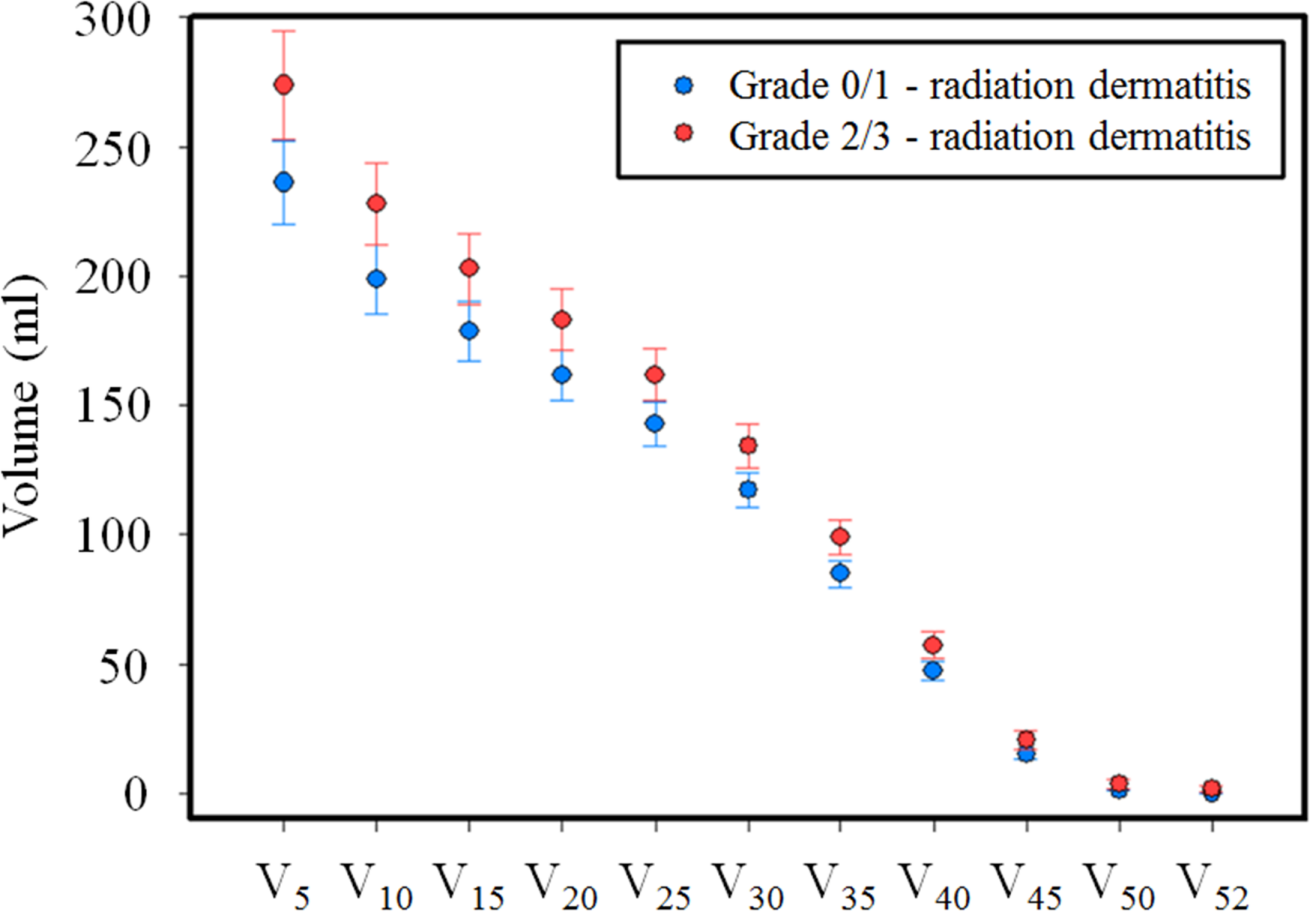
Comparison of the mean skin dose-volume histograms of patients with and without radiation dermatitis (RD) grade 2^+^ toxicity. Blue line = RD grade ≤ 1; red line = RD grade ≥ 2. V_X_ is the irradiation dose to the skin (5, 10, 15, 20, 25, 30, 35, 40, 45, 50, or 52 Gy).

The 95 RT plans used in this study achieved comparable PTV coverage, and the dose prescription policies were based on the percentage of the prescribed dose that covered >95% of the PTV (V95% ≥ 47.88 Gy) and spared sensitive structures similarly. An RD grade 1, 2, and 3 toxicity was observed in 43 (45%), 45 (48%), and seven patients (7%), respectively. The patients with RD grade 2^+^ toxicities were grouped into group 1 (n = 52); those without RD toxicities were grouped into group 0 (n = 43) (Table 1). Fig 1 shows a comparison of the mean dose-volume histograms (DVHs) for patients with and without RD grade 2+ toxicities. Most cases of RD grade 2+ toxicity occurred when a higher dose-volume was irradiated.

The initial dosimetric candidate predictive factors for the patients are shown in Table 2. The multicollinearity between the candidate factors and patients who suffered RD toxicities were excluded using LASSO. Table 3 shows the predictive factors for RD, ranked in descending order according to LASSO prediction processing in the multivariable logistic regression analysis. LASSO fitting of these dosimetric factors and patient characteristics led to the selection of four predictive factors: V_35 Gy_, energy, surgery and CI. The corresponding coefficients of the NTCP models for all the selected predictive factors are shown in Table 4. The NTCP model was S = –1.44 + (V_35 Gy_ × 0.05) + (energy × corresponding value) + (surgery × corresponding value) + (CI × –1.66). The coefficients and odds ratios of the NTCP models for the selected factors are shown in Table 5.

**Table 3:** Predictive factors correlation ranked by LASSO.

Rank	Factor	Rank	Factor	Rank	Factor	Rank	Factor
**1.**	V_35 Gy_	9.	PTV-V_105%_	17.	Smoking	25.	V_45 Gy_
**2.**	Energy	10.	Age	18.	Tumor Site	26.	V_50 Gy_
**3.**	Surgery	11.	V_40 Gy_	19.	IMN	27.	PTV-V_95%_
**4.**	CI	12.	V_30 Gy_	20.	BMI	28.	V_15 Gy_
**5.**	PTV-V_100%_	13.	PTV-V_107%_	21.	TV-V_110%_	29	V_20 Gy_
**6.**	V_5 Gy_	14.	Chemotherapy	22.	PTV (cm^3^)	30.	V_25 Gy_
**7.**	V_52 Gy_	15.	SCF	23.	V_10 Gy_	31.	
**8.**	AJCC	16.	TV-V_107%_	24.	HI	32.	

Abbreviations: LASSO: Least Absolute Shrinkage and Selection Operator; PTV: Planning Target Volume; BMI: body mass index; PTV-Vx% = percent volume receiving X% of prescribed dose within PTV. Treated volume (TV) = the tissue volume which received the prescribed dose. TV-Vx% = percent volume receiving X% of prescribed dose within TV; HI: Homogeneity Index; CI: Conformity Index; SCF: Supraclavicular fossa; IMN: Internal mammary lymph nodes; T-Boost: Tumor bed boost by electron; Vx was defined as skin volume received X Gy, and X was 5, 10, 15, 20, 25, 30, 35, 40, 45, 50, and 52 Gy at the selected steps.

**Table 4:** System performance evaluation.

Predictive factors	HL	SB-S	R^2^	Omnibus	AUC
**V**_**35 Gy**_	0.14	0.10	0.14	<0.01	0.69 (0.58–0.79)
**V**_**35 Gy**_**, Energy**	0.21	0.12	0.16	0.01	0.70 (0.60–0.81)
**V**_**35 Gy**_**, Energy, Surgery**	0.78	0.19	0.24	<0.01	0.75 (0.66–0.85)
**V**_**35 Gy**_**, Energy, Surgery, CI**	0.32	0.20	0.25	<0.01	0.76 (0.66–0.86)
**V**_**35 Gy**_**, Energy, Surgery, CI, PTV-V**_**100%**_	0.17	0.20	0.25	<0.01	0.76 (0.66–0.86)

H-L: Hosmer & Lemeshow test; SB-S: Scaled Brier Score; AUC: area under the curve;V_35_ was defined as skin volume received 35 Gy; CI: Conformity Index; PTV-Vx% = percent volume receiving X% of prescribed dose within PTV

**Table 5:** Multivariable logistic regression coefficients and odds ratios for the NTCP models for the RD toxicity after treatment.

Predictive factors	β	*p*-value	odds ratio	95% CI
**(n = 4)**				
**V**_**35 Gy**_	0.05	<0.01	1.05	1.02–1.08
**Energy**				
** 6X (0)**		0.25		
** 10X (1)**	-1.92	0.11	0.15	0.01–1.53
** 6X&10X (2)**	-1.07	0.30	0.35	0.05–2.58
**Surgery**				
** BCS (0)**				
** MRM (1)**	-1.03	0.07	0.36	0.12–1.10
**CI**	-0.44	0.40	0.65	0.24–1.78
**Constant**	-1.44	0.45	0.24	

*Abbreviations*: RD: radiation dermatitis; CI: Conformity Index; V_35_ was defined as skin volume received 35 Gy. Surgery: BCS: patients with breast conserving surgery; MRM: patients with modified radical mastectomy; 95% CI: 95% Confidence Interval

The overall performance of the NTCP model verified using Omnibus, scaled Brier score, and Nagelkerke R^2^ was satisfactory and corresponded well with the expected values in Table 4. The AUC for the NTCP model discrimination measure was ≥0.76. The Hosmer–Lemeshow test (calibration measure) showed a significant agreement between the predicted risk and observed outcome.

**Fig 2:**
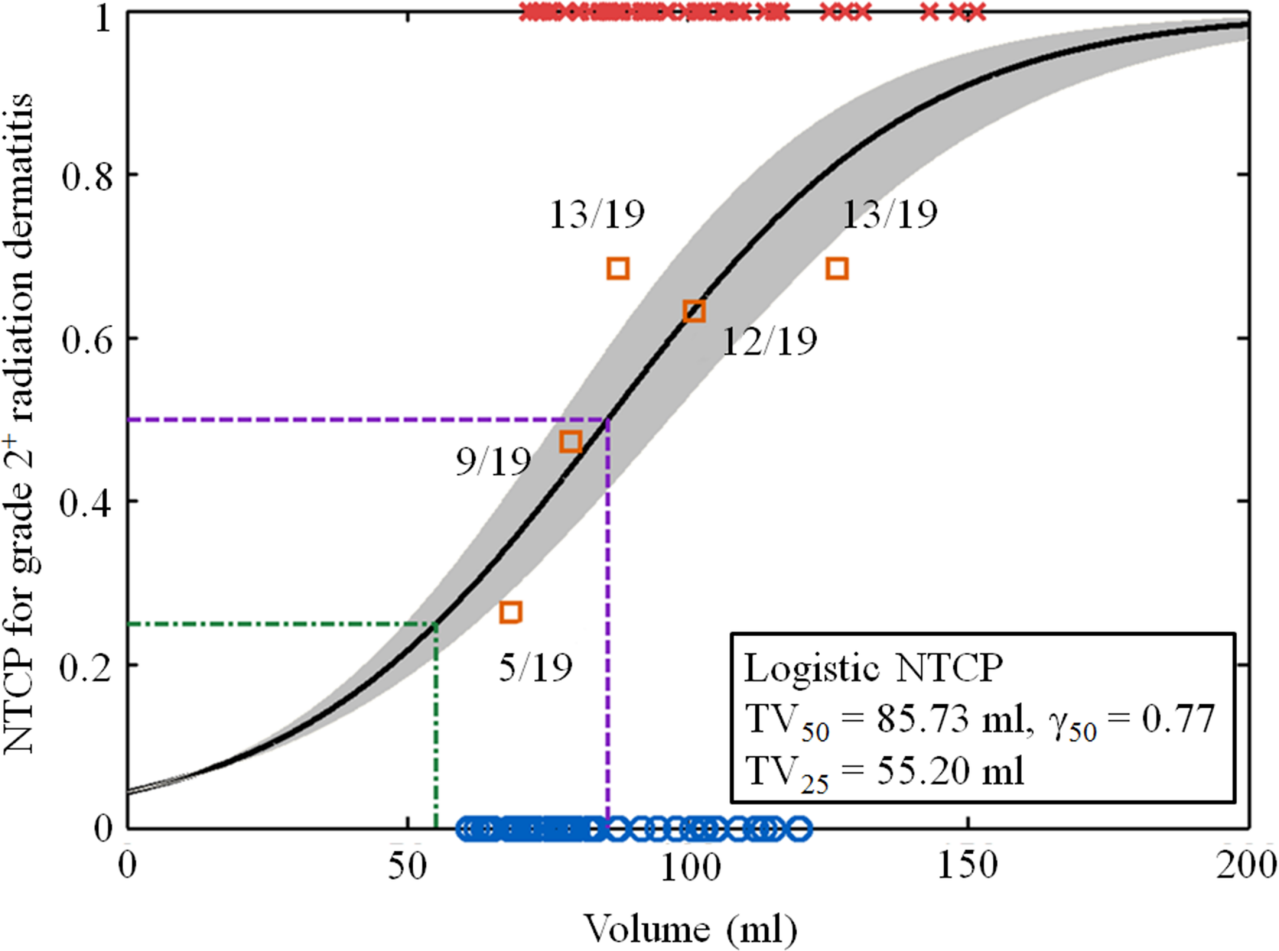
Probability model for normal tissue complications for skin dose of 35 Gy (V_35_). TV_25_ and TV_50_ are the tolerance volumes corresponding to complication rates of 25% and 50%, respectively, and γ_50_ is the normalized slope of the volume response curve.

V_35_ was the most significant dose volume parameter. The V_35_-fitted NTCP volume–response curve for the incidence of RD grade 2^+^ toxicity in breast cancer patients is shown in Fig 2. The parameters fitted were TV_50_ = 85.7 mL (confidence interval CI, 76.74–96.74), TV_25_ = 55.2 mL (CI, 49.40–62.20), and γ_50_ = 0.77 (CI, 0.35–1.26). The overall performance and calibration of the single-dose volume parameter V_35_-fitted NTCP model for grade 2^+^ RD toxicity tested using AUC, the Hosmer–Lemeshow test, and scaled Brier score were 0.69, 0.14, and 0.10, respectively.

## Discussion

Breast hybrid IMRT can deliver a more homogeneous dose distribution throughout the breast and efficiently avoids radiation hotspots [2]. This raises the probability that breast hybrid IMRT may significantly reduce RD toxicities. Freeman et al. (2009) [27] reported that 25% of breast cancer patients treated with three-dimensional conventional radiation therapy suffered grade 0/1 RD toxicities, whereas 75% experienced grade 2/3 toxicities. In the current study, 45% and 55% of patients experienced grade 0/1 and grade 2/3 toxicities, respectively. A similar report by Pignol et al. (2008) [28] demonstrated that breast IMRT could reduce ~15–20% of moist desquamation of the irradiated skin by delivering a more homogenous radiation dose through the breast and efficiently reducing hotspots. Chen et al. (2010) [29] also revealed that a PTV-V_107%_ > 28.6% and a TV-V_110%_ > 5.13% are two important predictors for RD. However, TV-V_110%_ and PTV-V_107%_ were not statistically significant in the current study, possibly because some radiation hotspots were removed using modern treatment techniques and fractionation schemes. There were only five patients with a PTV-V_107%_ > 28.6%, and four patients with a TV-V_110%_ > 5.13 in the current study. Despite the fact that breast hybrid IMRT technology allows a more homogenous radiation dose and results in fewer radiation hotspots, RD remains a significant problem. Therefore, the potential contribution of both potential predictive clinical factors and dosimetric information RD should be considered. Of the RD risk factors selected in the current study, CI showed a negative but not significant association with RD toxicity in hybrid IMRT breast cancer patients. A negative association means that better PTV coverage was achieved, but more RD toxicities were experienced. It is possible that a better PTV coverage leads to a higher dose of skin irradiation, as the skin near the PTV and the dose gradient were not sufficiently deep to avoid damage. This disadvantage is likely caused by the inability of photon-based RT to effectively limit the exposure of nearby organs; therefore further investigations are needed.

Trott et al. (2012) [30] stated that NTCP models are mostly based on simplified empirical models and consist of dose distribution factors that are possibly mixed with clinical- or treatment-related parameters. Different mechanisms are related to different dose effects and associations between the dose-per-fraction, dose rate, and treatment time duration and effects. They revealed that an NTCP model designed to accommodate the specific OAR should be developed for each side effect. In the current study, four risk factors were selected using LASSO with cross-validation: namely, the percentage of the skin volume that received >35 Gy (V_35Gy_), energy, surgery, and CI. The V_35Gy_ was the most significant dosimetric predictive factor for RD in hybrid IMRT breast cancer patients. A univariate point V_35Gy_ NTCP model was built for skin RD toxicity. The current data suggest that the skin volume that receives >35 Gy should be limited to <83.3 mL to keep the incidence of grade 2^+^ RD toxicity <50% in breast cancer patients receiving hybrid IMRT.

Energy and surgery were selected as initial predictive patient characteristics, but there were no significant differences between groups. The selection of energy was based on the patient chest-wall separation or the volume of the PTV; a larger PTV resulted in a larger energy selection. The mean PTV for the different energies selected were: 6X, 402.8 mL; 6X and 10X, 702.0 mL, and 10X, 738.4 mL. The purpose of energy selection is to reduce hotspots in the axillary area. Sun et al. (2013) [9] reported that the axillary or inframammary fold areas are the most common sites for moist desquamation.

Regarding breast cancer surgery (BCS and MRM), 60% of patients that underwent BCS and 49% of those receiving MRM had a risk of RD toxicity; the risk in the BCS group was greater than that in the MRM group. Because the mean PTV of BCS patients is larger than that of MRM patients (790 mL versus 580 mL, respectively), a larger PTV may increase the incidence of RD.

There was no association between breast size and the risk of RD among the candidate predictive factors used in the current study. A similar report by Freedman et al. (2009) [27] revealed that the degree of acute desquamation was greater in conventionally treated patients than IMRT-treated patients. Also, subgroup analyses revealed that breast IMRT was associated with a significant decrease in the maximum severity of RD compared with conventional radiation, regardless of breast size. In contrast, Vicini et al. (2002) [31] showed that breast volume was a significant contributing factor to RD in breast cancer patients after RT. In the current study, the mean PTV in groups 0 and 1 was 632 mL (3/43 patients had a breast volume > 1000 mL), and 739 mL (11/52 patients had a breast volume > 1000 mL), respectively. There was no statistically significant difference between the two groups, and most patients had a breast size < 1000 mL. Therefore, subgroup analysis did not identify breast volume as a significant factor (PTV was defined as breast size).

Patients receiving conventional chemotherapy (e.g. anthracyclines or taxanes) or targeted anticancer therapy with endothelial growth factor receptor (EGFR) inhibitors are at increased risk of developing severe RD [32]. However, in the current study, patients who had received chemotherapy did not exhibit an increased risk of RD. The first reason for this discrepancy is differences in the treatment regimens used. Anthracyclines and taxanes are not routine treatment options according to the current treatment guideline in our hospital. Furthermore, EGFR inhibitors are not used in breast cancer patients. The second reason is the chemotherapy and radiation therapy schedules used. Patients started their radiotherapy treatment 2 months later than chemotherapy for radiation therapy preparation and treatment planning. The chemotherapy effect had reduced by the time radiotherapy was initiated and patients had recovered sufficiently during in the duration of this time Therefore, the influence of chemotherapy was minimized and it was not a significant predictive factor in this study; there was no association between chemotherapy and the risk of RD.

Patient age was not a significant predictive factor for determining the severity of RD. Previous publications revealed that there was no evidence to suggest that elderly patients were more sensitive to irradiation [33–35], which is consistent with the observations in the current study.

Fisher et al. (1986) and Hälg et al. (2012) showed that the blood vessels in the skin run within the first 5 mm below the epidermis [36, 37]. Van Limbergen et al. (1990) reported the importance of sparing the terminal branches of the skin microvessels that lie 3 mm beneath the skin surface [38]. Fisher et al. (1986) noted that if the skin volume does not form part of the PTV, recurrence is rare in spite of the probability of recurrence [36]. In practice, not only should care be taken with regard to sparing the terminal branches of the skin microvessels, but consideration should also be given to avoiding the probability of recurrence. Therefore, we decided to clip the first 3 mm from the skin surface in our skin volume.

Thermoplastic casts were used to provide good fixation and reproducibility for breast cancer patients undergoing radiation therapy, but the disadvantage of using an immobilization cast is that it increases the surface dose to the breast via the bolus effect [39, 40]. However, the fact that our report did not take this effect into consideration is one of the drawbacks of this study. In practice, our patients were treated with the same type of thermoplastic cast, so the dose effect is assumed to be similar for each patient. However, this limitation needs to be investigated in the future.

The current study has several weaknesses, such as its retrospective study design and a relatively small population size and event number. These factors may have limited the statistical power. Additionally, as cited in a previous study, there is no gold standard for the measurement or management of RD and no standard, accurate scoring system for RD toxicity [10]. Moreover, the major limitation of this study is that it does not provide experimental verification of the calculated skin dose; however, such calculation remains challenge not only for most of the commercially-available treatment planning systems but also in phantom tests [41]. Kry et al. (2012) showed that the average magnitude of the local difference between the calculated and measured doses was 22% [41]. Skin doses are also not generally intuitive compared to doses throughout the rest of the body and are difficult to measure [41]. As such, in this study, we showed that evaluation of the relationships between the treatment factors and the RD toxicity during routine radiotherapy with certain procedures is a feasible option. Despite the lack of a precise measurement of the skin irradiation dose, our study provides a direct and convenient method to ascertain the relationship between DVHs and RD treatment toxicity.

## Conclusions

The predictive risk factors selected by the LASSO NTCP model are useful for further optimizing hybrid IMRT for RD toxicity. The most important predictive risk factors identified in the current study will help spare the skin and reduce toxicity as much as possible. RD complications decrease both the quality of life and compliance of breast cancer patients in RT. Careful RT planning can identify dosimetric issues in PTV coverage and promote OAR sparing. The current study found that V_35Gy_ could be applied to predict the risk of grade 2^+^ dermatitis in breast cancer patients after RT. We suggest a dose-volume constraint for the volume of skin that may be irradiated in breast cancer patients. Namely, the skin volume receiving a dose >35 Gy should be limited to <85.7 mL to keep the incidence of RD grade 2^+^ toxicity < 50%. Moreover, the volume of skin receiving a dose >35 Gy should follow the sparing tolerance, and patient characteristics should be considered to avoid RD toxicity. However, one more issue has to be mentioned, i.e. for the commercial TPS systems, the typical dose grid size to commission the software system is about 3 mm which is comparable to the thickness of the skin. This limitation still exists currently; when the dose grid effect can be overcome by the new technology or algorithms, the result and the system performance can be improved.

## Supporting information

S1 File Data underlying this study. (XLSX)

## Abbreviations

IMRT: intensity modulation radiation therapy
LASSO: least absolute shrinkage and selection operator
NTCP: normal tissue complication probability
RD: radiation dermatitis
BCS: breast conserving surgery
MRM: modified radical mastectomy
CT: computed tomography
TPS: treatment planning system
DMPO: direct machine parameter optimisation
gEUD: generalised equivalent uniform dose
CCC: collapsed cone convolution
CTV: clinical target volume
PTV: planned target volume
OAR: organs at risk
SCF: supraclavicular fossa
IMN: internal mammary lymph nodes
NCCN: National Comprehensive Cancer Network Clinical Practice Guidelines
CMF: cyclophosphamide methotrexate fluorouracil
CEF: cyclophosphamide epirubicin 5-fluorouracil
RTOG: Radiation Therapy Oncology Group
BMI: body mass index
TV: treated volume
CI: conformity index
